# Clinical Efficacy of a Spiral CT-Guided Balloon Compression Day-Surgery Operation for the Treatment of Trigeminal Neuralgia

**DOI:** 10.3389/fneur.2022.923225

**Published:** 2022-07-06

**Authors:** Bing Xu, Zi-pu Jia, Hao Ren, Lan Meng, Ying Shen, Tao Wang, Fang Luo, Rui Lv

**Affiliations:** ^1^Department of Radiology, Beijing Tiantan Hospital, Capital Medical University, Beijing, China; ^2^Department of Anesthesiology, Beijing Tiantan Hospital, Capital Medical University, Beijing, China; ^3^Department of Pain, Beijing Tiantan Hospital, Capital Medical University, Beijing, China; ^4^Department of Neurosurgery, Beijing Tiantan Hospital, Capital Medical University, Beijing, China

**Keywords:** trigeminal neuralgia, day-surgery, efficacy, balloon compression, radiology

## Abstract

**Objective:**

This study aimed to investigate the clinical efficacy of a balloon compression day-surgery operation under the guidance of spiral computed tomography (CT) three-dimensional (3D) reconstruction for the treatment of trigeminal neuralgia.

**Methods:**

The clinical efficacy and related indexes of 380 patients with trigeminal neuralgia treated by a spiral CT-guided balloon compression day-surgery operation in the pain department of Beijing TianTan Hospital, from October 2017 to March 2021, were retrospectively analyzed.

**Results:**

Five patients failed due to foramen ovale puncture or in placing the balloon; two patients had ineffective results after the operation and re-entered the hospital for secondary balloon compression. The initial effective rate of the operation in the 380 patients was 98.16%. All patients were discharged on the day of the operation, the average operation time was 26.46 ± 12.15 min, and the average interval from the completion of the operation to discharge was 2.67 ± 0.95 h. During the follow-up period (1–41 months), 12 patients had pain recurrence, and a Kaplan–Meier analysis revealed that the cumulative pain-free recurrence survival rate at 41 months after the operation was 80.64%. No complications related to foramen ovale puncture occurred.

**Conclusion:**

The spiral CT-guided balloon compression day-surgery operation is safe, effective, and worthy of clinical promotion.

## Introduction

Trigeminal neuralgia is a common neuropathic severe pain in the facial trigeminal innervation area. The annual incidence of trigeminal neuralgia is ~0.03–0.05%. The incidence in the elderly population is high, as patients over 40 years old account for 70–80% of the total patients (1). The quality of life of patients with trigeminal neuralgia who fail to respond to conservative treatment is seriously affected. Intractable trigeminal neuralgia is gradually becoming one of the primary disease burdens for the elderly. As China's population continues to age, the number of patients with trigeminal neuralgia increases every year, and as our department specializes in the diagnosis and treatment of painful diseases, the number of patients with trigeminal neuralgia will likely increase.

According to the International Classification of Headache Disorders 3rd Edition (ICHD-3), trigeminal neuralgia is divided into three types: classic, secondary, and idiopathic (2). Because the pathogenesis of trigeminal neuralgia is not completely clear, there is a lack of ideal etiological treatment. For patients with trigeminal neuralgia who still have poor control of pain symptoms or drug-related side effects that are not tolerated after carbamazepine and oxcarbazepine treatment, surgery is usually chosen. For patients with classic trigeminal neuralgia with offending vessel compression, craniotomy microvascular decompression or microvascular decompression combined with the partial transection of the trigeminal sensory root is feasible. Patients with trigeminal neuralgia, who fail to respond to conservative treatment, can be treated with percutaneous microballoon compression (PMC) of the semilunar ganglion, radiofrequency thermocoagulation, γ knife radiosurgery (3–6), and other neurodestructive treatments. PMC is minimally invasive, safe, and effective. The routine treatment for PMC, in China and abroad, is performed in the hospital, and the fastest discharge time reported in the literature is the second day after the operation (7).

With the improvement of medical technology and management levels, day-surgery operations have developed rapidly (one in which the patient is admitted, operated on, and discharged within one working day). Day-surgery operations greatly shorten the hospitalization time of patients, reduce the hospital-acquired infection rate, speed up the turnover of beds, and improve the utilization rate of medical resources; all of these results (especially from minimally invasive day-surgery operations), in turn, reduce medical costs. In 2017, the pain department of our hospital took the lead in performing day-surgery PMC for trigeminal neuralgia under general anesthesia and guided by spiral computed tomography (CT) three-dimensional (3D) reconstruction images at home and abroad. Our hospital preliminarily reported that 22 patients received day-surgery PMC in the early stage, all of whom achieved satisfactory treatment results (8). Therefore, it was suggested that a spiral CT-guided PMC day-surgery operation of the semilunar ganglion was a safe, effective, and feasible method to treat trigeminal neuralgia. Subsequently, the pain department of our hospital performed hundreds of PMC day-surgeries. Here, the clinical efficacy of more patients is reported.

## Methods

### General Information of Patients

The ethics committee of Beijing TianTan Hospital approved this study. In view of the retrospective nature of this study, informed consent of the patients was exempted. The data were collected and analyzed retrospectively from the medical records of Beijing TianTan Hospital and from the routine post-operative follow-up data of the patients in the pain department.

#### Inclusion Criteria

(1) The age of the patient was >18 years old; (2) The patients met the diagnostic criteria of trigeminal neuralgia defined by the ICHD-3; (3) Patients underwent day-surgery PMC of the trigeminal semilunar ganglion guided by spiral CT 3D reconstruction under general anesthesia.

#### Exclusion Criteria

Patients with incomplete perioperative clinical data or follow-up data.

### Treatment Method

#### Pre-Operative Preparation

The pre-operative examinations were performed (i.e., a routine blood test, liver and kidney function, blood electrolytes, coagulation function, chest X-ray films, and an electrocardiogram (ECG). For patients whose pre-operative examination results were abnormal and needed to delay the day-surgery operation, a new appointment was made for the operation after correcting the abnormal indexes. A pre-operative evaluation was conducted to determine whether the conditions of the patients with hypertension, diabetes, coronary heart disease, cerebrovascular disease, and other complications were stable. Patients with unstable complications were transferred to the corresponding specialty for treatment. After the complication became stable, the patients went to the pain department for reevaluation and rescheduling of the day-surgery operation.

#### Anesthesia

The patient lay on their back on the scanning table in the CT operating room, with their head in a natural median position. The patient's blood pressure, heart rate, ECG, and blood oxygen saturation were routinely monitored. A peripheral vein of the upper limbs was opened, and 0.01 mg/kg of atropine was given intravenously before the operation. Anesthesia was induced by sequential intravenous administration of 0.2 μg/kg of sufentanil, 1.5–2 mg/kg of propofol, and 0.1 mg/kg of Cisatracurium, and a suitable laryngeal mask was placed. Patients with poor alignment of the laryngeal mask received endotracheal intubation. Anesthesia was maintained by a continuous intravenous pumping of propofol at 4–6 mg/(kg h) and remifentanil at 0.05–0.1 μg/(kg min) with a micropump, and the drug dosage was adjusted according to the circulation of the patients during the operation.

#### Surgery

**Figure 1:**
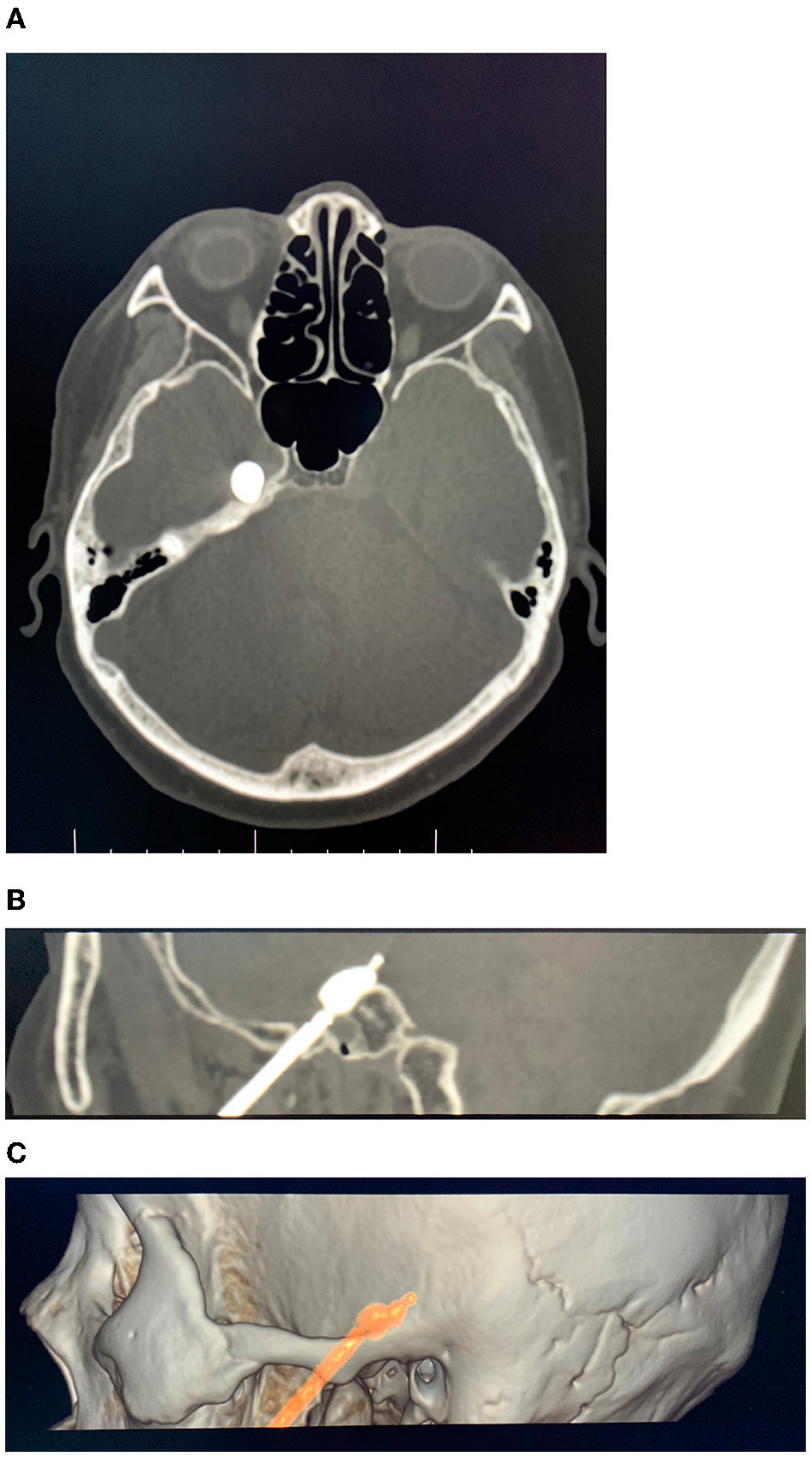
Axial **(A)** and sagittal **(B)** computed tomography (CT) reconstruction images showing the balloon shape and position were correct (the white arrow points to the balloon). **(C)** Entry point and target direction.

**Figure 2:**
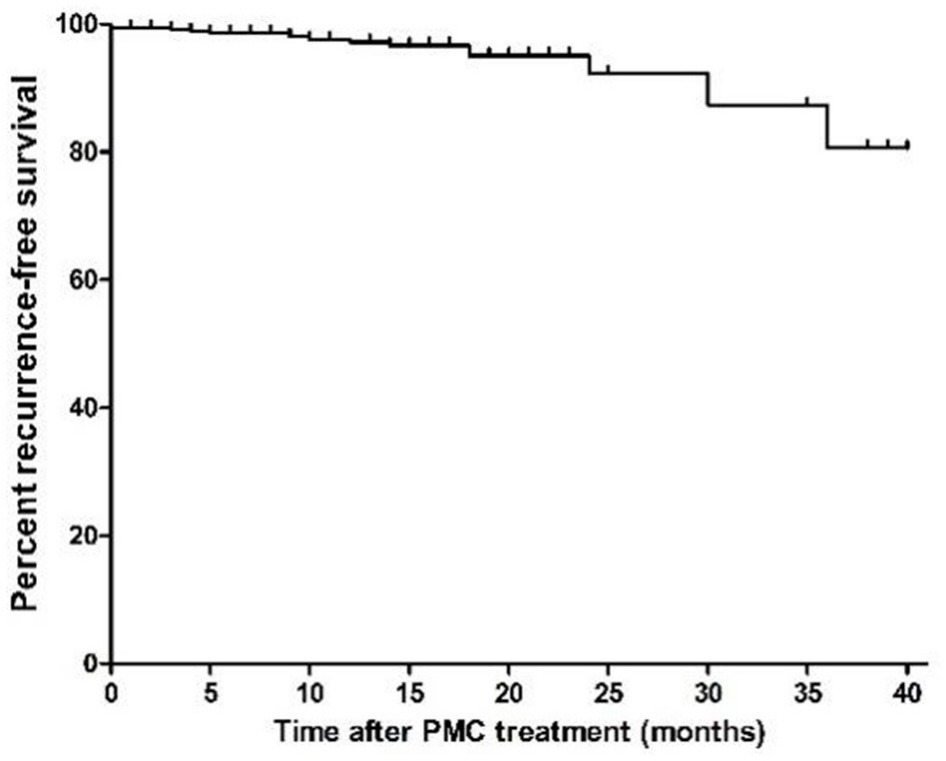
Kaplan–Meier recurrence-free survival curves for patients who underwent percutaneous microballoon compression (PMC) treatment.

After routine disinfection, a surgical drape and towel were place. Local infiltration anesthesia was induced at the puncture site and along the puncture route by 0.5% lidocaine. Surgery using Hartel landmarks approach for the puncture. According to the clinical experience of the surgeon, a 14G blunt head puncture probe (CTZ-15, Shenzhen Shineyard Medical Device Co. Ltd., Shenzhen, China) was inserted and placed near the foramen ovale of the skull base. Thin-slice CT scanning of the skull base was performed (GE Lightspeed 64-slice CT machine, USA). According to the relative spatial position relationship between the puncture probe and the foramen ovale in the spiral CT 3D reconstruction image (GE AW VolumeShare 2 workstation, aw4.4 version, USA), the operator adjusted the puncture probe until it successfully entered the foramen ovale (Figure 1). The needle core was withdrawn, and the aspirated disposable balloon catheter for brain surgery (QKS-1850567, Shenzhen Shineyard Medical Device Co. Ltd., Shenzhen, China) was placed into the probe needle, after which the Meckel's balloon was placed. The guidewire was withdrawn, and 0.3–0.5 ml of non-ionic contrast medium (Omnipaque) was slowly injected into the balloon. The position and shape of the balloon were confirmed with the aid of the spiral CT 3D reconstruction image (Figure 2); when the balloon was pear shaped, the position was accurate. According to the surgeon's experience, 0.5–1.0 ml of contrast medium was injected and compressed for 3–4 min. Next, the balloon was evacuated and withdrawn together with the probe needle (8). Sterile dressing was applied after 5 min of pressure and hemostasis at the puncture point.

#### Post-Operative Management

After the operation, the laryngeal mask or endotracheal tube was removed from the patient, who returned to the post-anesthesia recovery room for observation until discharge. The discharge criteria of the patients after PMC included the following items: (1) The patient had stable vital signs, lucid mental state, and clear verbal expression; (2) No dizziness, nausea, and vomiting were present; (3) The patient's walking ability was restored to the same level as before the operation. If the discharge standard could not be met on the day of operation, the patient was transferred to the ward and discharge was delayed. After discharge, the patients received comprehensive post-operative nursing guidance through the Wechat platform of the pain department. If the patient's pain was not relieved 1 month after the operation, they went to the clinic. If the patient's ongoing pain was considered to be incomplete trigeminal nerve damage, another PMC operation was arranged.

### Data Acquisition and Analysis

The pre-operative, intraoperative, and post-operative clinical data were collected from the inpatient medical records and the pain department follow-up database. The pre-operative data included the patient's gender, age, pre-operative course of disease, trigeminal neuralgia side, affected trigeminal nerve branches, pre-operative Barrow Neurological Institute (BNI) pain rating, and previous surgical treatment. The intraoperative data included the operation time, anesthesia time, volume of contrast medium injected into the balloon, time of balloon compression, and intraoperative trigeminal inhibitory reaction. The time from the completion of the operation to patient discharge was recorded. To improve the medical quality of care, the pain department followed up with every patient immediately after the PMC operation. Initial follow-up based on initial clinical efficacy evaluation took place 1 week after PMC treatment by the surgeons who performed the operation. Long-term follow-ups were carried out 1 month after the operation, local patients were followed up with an outpatient department, and foreign patients were followed up by telephone. Subsequently, all patients received intermittent telephone follow up every month for at least 1 year, every 3 months for the next 2 years, and thereafter every 12 months by specially trained investigators on schedule. Details of pain relief, recurrence, complications, and side effects were recorded in the database during each follow-up conversation. The BNI pain rating, complications, and side effects (e.g., facial numbness, masticatory muscle weakness, vision loss, diplopia, herpes, hearing loss, keratitis, corneal ulcer, and cerebrospinal fluid leakage) were recorded.

### Assessment Criteria

The patients' pain intensity was rated by the BNI scale (I: no trigeminal neuralgia, no need of drug treatment; II: occasional pain, no need of drug treatment; III: some pain, which can be controlled by drugs; IV: some pain, which cannot be completely controlled by drugs; and V: severe pain or no pain relief). Pain relief in the immediate post-operative period is defined as a BNI pain rating of grade I or II after awakening from anesthesia. Preliminary post-operative pain relief is defined as a BNI pain rating of grade I or II 1 month after the PMC operation. The effective rate = (number of cases at BNI I + II)/total number of cases ×100%. The pain recurrence is defined as an increase in the post-operative BNI pain intensity rating from grade I or II to grade III or higher.

### Statistical Analysis

The SPSS 20.0 statistical software was used for the statistical analysis of each collected variable. For the measurement data, if the variables were normally distributed, the mean and standard deviation were calculated, and an inter-group comparison was conducted using a *t*-test or an analysis of variance. The non-normally distributed data were expressed as the median and interquartile range (*IQR*) and were compared between the two groups using the Mann–Whitney *U*-test or the Kruskal–Wallis *H*-test. The categorical data were expressed as a frequency and a percentage and were compared between the two groups using a chi-square test. The Kaplan–Meier method was used to evaluate the pain-free recurrence survival rate of patients.

## Results

### General Data of Patients Before Operation

A total of 380 patients with trigeminal neuralgia who underwent a spiral CT 3D reconstruction day-surgery PMC operation of the trigeminal semilunar ganglion in the pain department of Beijing TianTan Hospital, from October 2017 to March 2021, were included in this analysis. Among these 380 patients, 167 patients were men, and 213 patients were women. The age of these patients was 72.40 ± 8.28 years old, the course of the disease was 8.33 ± 7.21 years, 127 patients had left pains, and 253 had right pains. Eleven patients had pain in the first branch, 44 had pain in the second branch, 79 had pain in the third branch, 81 had pain in both the first and second branches, 121 had pain in both the second and third branches, five had pain in both the first and third branches, and 39 had pain in all of the branches (the first, second, and third). Nine patients had trigeminal neuralgia secondary to intracranial tumors, seven of which had undergone intracranial tumor resection. Thirty-eight patients had undergone craniotomy microvascular decompression or microvascular decompression combined with the partial transection of the trigeminal sensory root, 55 patients had undergone radiofrequency thermocoagulation, 48 patients had undergone γ knife treatment, and six patients had a previous PMC surgery.

### Effect of the Day-Surgery PMC Operation

Among the 380 patients, 379 patients completed the operation under general anesthesia and laryngeal mask ventilation; one patient (0.3%) had poor alignment after the laryngeal mask placement and required endotracheal intubation ventilation.

Failure to puncture the foramen ovale (due to a narrow foramen ovale) occurred in one patient (0.3%). Success in puncturing the foramen ovale during the operation, but a failure in the balloon implantation, occurred in two patients (0.5%). Effective compression was not achieved in two patients (0.5%) because the balloon ruptured three times when they were injected with the contrast agent. A total of five patients (1.3%) did not complete the PMC surgery, and radiofrequency thermocoagulation was completed during the operation.

The successful puncture of the foramen ovale occurred in 375 patients (98.7%), along with correct balloon position and shape (a pear shape) under the guidance of spiral CT 3D reconstruction image. Pain relief in the immediate post-operative period (BNI I or II) was reported by 368 patients (96.8%), and 373 patients (98.2%) had preliminary post-operative pain relief (1 month after operation). Two patients (0.5%) had no facial hypoesthesia and hypoalgesia at 1 month after the operation; however, after hospitalization, their pain was relieved after they underwent a secondary PMC with an appropriate increase of balloon volume and an extension of the compression time.

The dosage of contrast agent injected into the balloon was 0.76 ± 0.12 ml, the time of the intraoperative balloon compression was 4.14 ± 1.01 min, the median time of the PMC operation was 25 min (IQR: 18–30 min), the median time of anesthesia was 35 min (IQR: 30–45 min), and the average interval from the completion of operation to patient discharge was 2.67 ± 0.95 h. All patients were smoothly discharged on the day of operation, and none of these patients delayed discharge or were transferred to the general ward.

Of the 373 patients who achieved effective results after the PMC day-surgery operation, the follow-up time ranged from 1 month to 41 months, with a median of 14 months. Pain recurrence occurred in 12 patients; the Kaplan–Meier curve is shown in Figure 2. The Kaplan–Meier analysis revealed that the 12 patients relapsed at 3, 4, 5, 9, 10, 12, 14, 18, 18, 24, 30, and 36 months after the PMC operation, respectively. At 12, 24, 36, and 41 months after the operation, the cumulative pain-free recurrence survival rates were 97.16, 92.22, 80.64, and 80.64%, respectively.

### Complications and Side Effects of the PMC Day-Surgery Operation After General Anesthesia

When patients wake up and back to Post-anesthesia care unit (PACU), 15 patients (3.95%) suffered from post-operative dizziness. Forty-five patients (11.84%) reported nausea and 21 of them (5.53%) suffered from vomiting after the PMC operatin. All these side effects mentioned above disappeared within 4 h after treatment. Three patients (0.79%) suffered from post-operative delirumin which disappeared 2 days later. Among the 375 patients who completed the foramen ovale puncture and balloon placement, no serious complications related to the foramen ovale puncture occurred. No severe cardiac arrest occurred in any patient, and 191 patients (50.9%) had different degrees of trigeminal inhibition during operation. After the operation, 362 patients (96.5%) had different degrees of facial numbness on the affected side, and 293 patients (78.1%) had masseter weakness on the affected side. After the operation, 243 patients (64.8%) developed ipsilateral facial herpes simplex that subsided after 1–3 weeks. Diplopia occurred in five patients (1.3%) and recovered 1–3 months after the operation, and keratitis occurred in 6 patients (1.6%), but it improved after symptomatic treatment. No serious complications (e.g., arteriovenous fistula, intracranial hemorrhage, cerebral infarction, corneal ulcer, and cerebrospinal fluid leakage) occurred in any patient.

## Discussion

Since 2017, the pain department at our hospital successfully performed day-surgery PMC of trigeminal semilunar ganglion guided by spiral CT 3D reconstruction image under general anesthesia and laryngeal mask for the first time. This study reported, for the first time, the clinical effect of day-surgery PMC for 380 patients at home and abroad.

First, this study revealed that, consistent with the success rate reported in previous routine inpatient surgeries (9), the success rate of day-surgery operations was as high as 98.2%. One of the reasons for the high success rate was that all operations were completed under the guidance of advanced spiral CT 3D reconstruction technology. Currently, the majority of PMC surgeries at other hospitals are completed under the guidance of two-dimensional X-ray images (e.g., a c-arm X-ray). It was reported in previous literature that the success rate of CT-guided foramen ovale puncture applied in radiofrequency thermocoagulation in the treatment of trigeminal neuralgia was higher than that of conventional X-ray-guided puncture (10–12). Huo et al. (13) found that compared with c-arm guided surgery, CT-guided PMC surgery has the following advantages: (1) the FO can be better visualized independently of the patient's position; (2) needle correction or insertion can be performed much more easily because of the direct fluoroscopic control; and (3) the needle position, balloon position, balloon configuration, and the volume of the inflated balloon is more reliably determined. The use of Dyna-CT-assisted PMC has a low incidence of complications and a good prognosis. Xiao et al. (14) reported the comparison of CT-guided and C-arm guided PMC surgery. The 3D-CT group required less time than the C-arm group for puncture (*p* < 0.001) and for the whole operation (*p* < 0.001). In our study, puncture of foramen ovale under the guidance of spiral CT can reduce puncture damage and shorten puncture time to a certain extent. After the puncture is in place, the spiral CT 3D reconstruction images accurately determine the position and shape of the balloon from the coronal, sagittal, axial, and 3D images, ensuring the success of the balloon compression.

This study also reported that the total average operation time was only 39.23 min shorter than Lin et al. (15), and Aydoseli et al. reported that the average time for the same operation guided by neuronavigation was 68.2 and 43 min (16). Neuronavigation technology is a more advanced intraoperative real-time visualization technology. However, neuronavigation requires higher-cost equipment, and during the operation, the skull needs to be fixed on the head frame. In addition, the shape of the balloon cannot be verified during the operation. Although 3D CT can only provide semi-real-time image guidance, it is very helpful to be able to confirm and correct the position of the puncture needle and the balloon. Another advantage of CT guidance is that clinicians avoid radiation exposure. In addition, the operation technique is improved, the average operation time is shortened, and the anesthesia time is reduced, avoiding the accumulation of anesthetic drugs in the patients so that they recover quickly after the operation. This is necessary to ensure that the patient is discharged on the day of the operation.

In this study, all the patients under general anesthesia, recovered and were discharged with no issues on the day of operation. No patients were transferred to the inpatient department due to serious post-operative complications, and after discharge, there were no patients with serious complications leading to readmission. In recent years, Chinese scholars have also proposed that (17, 18) PMC surgery can be completed under conscious sedation, analgesia, and the monitoring of vital signs. Theoretically, PMC surgery has less physiological interference to patients under conscious sedation and analgesia. However, whether general anesthesia or conscious sedation and analgesia have more of an advantage for patients undergoing PMC should be studied *via* prospective controlled research in the future.

Third, this study revealed that PMC day-surgery operations ensured patient safety by improving the safety of the operation and by avoiding serious complications requiring hospitalization during and after the operation. These were necessary conditions to ensure that the patients were quickly discharged. In a survey conducted in Scotland, only 18% of trained neurosurgeons believed that they could independently complete the puncture and catheterization of the foramen ovale (19). Due to unsuccessful puncture, incorrect position of the puncture needle, or repeated attempts, patients may have rare but serious complications, such as carotid-cavernous sinus fistula, dural arteriovenous fistula, intracranial hematoma, cerebrospinal fluid rhinorrhea, central nervous system infection, cranial nerve injury, or even death (13, 16, 20). In this study, no serious complications related to the puncture of the foramen ovale occurred in any of the 380 patients. This is closely related to the use of advanced imaging technology.

In recent years, clinical studies generally believe that microballoon compression, radiofrequency thermocoagulation, and microvascular decompression in the treatment of trigeminal neuralgia can achieve significant therapeutic effects in the short term. The operation of PMC is simple, avoids the risk of craniotomy, and is suitable for patients with underlying diseases, poor physical conditions, and unable to tolerate craniotomy (21). Clinically, MVD can be the first choice for young patients with good physical condition and normal cardiopulmonary function to reduce the occurrence of post-operative complications. However, PMC is recommended for patients who are old and weak and have underlying diseases and cannot tolerate craniotomy. Zhao et al. (22) compared the efficacy and safety of radiofrequency thermocoagulation and PMC in the treatment of primary trigeminal neuralgia, and found that the hemodynamics of patients in the PMC group were more stable and the operation time was shorter than that in the radiofrequency thermocoagulation group. The incidence of BNI facial numbness and masseter weakness at 24 h after surgery was higher in the radiofrequency thermocoagulation group than in the balloon compression group, and the incidence of corneal reflex inertia at 3 months after surgery was higher than in the balloon compression group.

Of course, consistent with the conclusions of other studies (23, 24), PMC surgery is a neurodestructive treatment and will inevitably have side effects (e.g., facial numbness and masticatory muscle weakness). These side effects were gradually alleviated and tolerated in most patients after the operation. Some researchers believe that shortening the compression time of the balloon may reduce the incidence of complications; however, insufficient compression time may lead to failure of pain relief or to pain recurrence. Wang et al. (20) reported that a shortened time and repeated compression PMC technique had similar curative effects to traditional continuous compression, but that patients' facial numbness was less. To date, there is no consensus on the appropriate compression time and balloon volume for PMC surgery. Future research should explore individualized balloon compression techniques (e.g., intraoperative monitoring of balloon pressure) to provide objective indicators for PMC surgery (25) instead of relying on personal experience.

## Conclusion

The spiral CT-guided balloon compression day surgery for trigeminal neuralgia has the advantages of short operation time, high safety, low incidence of post-operative serious complications and adverse reactions, and high pain relief rate, which is worthy of promotion.

## Limitation

The present study does have some limitations. Because it was a retrospective study, the related indicators of the conventional c-arm X-ray guided surgery were not evaluated and compared (e.g., foramen ovale punctures failure rate, operation time, and others). This study only collected the clinical data from October 2017 to March 2021, so there was a lack of long-term follow-up results. Due to the retrospective nature of this study, some valuable variables (e.g., balloon pressure) were not obtained; therefore, a prospective randomized controlled study on the efficacy and safety of multi-parameter PMC in the treatment of trigeminal neuralgia should be performed. This study did not compare the health economic indicators of PMC day-surgery operations and inpatient surgery in the treatment of trigeminal neuralgia; this should be done in the future to clarify the value of day-surgery operations in reducing the economic burden of patients and the proportion of national medical insurance reimbursement.

## Data Availability Statement

The original contributions presented in the study are included in the article/supplementary material, further inquiries can be directed to the corresponding author.

## Ethics Statement

The studies involving human participants were reviewed and approved by Beijing Tiantan Hospital, Capital Medical University. The patients/participants provided their written informed consent to participate in this study.

## Author Contributions

BX, Z-pJ, and FL: conception and design of the research. Z-pJ, HR, BX, and RL: acquisition of data. LM, YS, FL, and TW: analysis and interpretation of the data. Z-pJ: statistical analysis. BX, Z-pJ, and RL: writing of the manuscript. FL and RL critical revisd of the manuscript for intellectual content. All authors read and approved the final draft.

## Conflict of Interest

The authors declare that the research was conducted in the absence of any commercial or financial relationships that could be construed as a potential conflict of interest.

## Publisher's Note

All claims expressed in this article are solely those of the authors and do not necessarily represent those of their affiliated organizations, or those of the publisher, the editors and the reviewers. Any product that may be evaluated in this article, or claim that may be made by its manufacturer, is not guaranteed or endorsed by the publisher.

## References

[1] Wang CH Zhao R Ran DW. The progress in diagnosis and treatment of trigeminal Neuralgia. J Clin Neurol. (2019) 32:390–3. 10.3969/j.issn.1004-1648.2019.05.030

[2] Headache Classification Committee of the International Headache Society (IHS). The International Classification of Headache Disorders. 3rd edition. Headache Classification Committee of the International Headache Society (2018). 10.1177/0333102417738202 29368949

[3] Alshukry A Salburgo F Jaloux L Lavieille JP Montava M. Trigeminal neuralgia (TN): a descriptive literature analysis on the diagnosis and management modalities. J Stomatol Oral Maxillofac Surg. (2017) 118:251–4. 10.1016/j.jormas.2017.06.010 28652174

[4] Wang DD Raygor KP Cage TA Ward MM Westcott S Barbaro NM. Prospective comparison of long-term pain relief rates after first time microvascular decompression and stereotactic radiosurgery for trigeminal neuralgia. J Neurosurg. (2017) 24:1–10. 10.3171/2016.9.JNS16149 28298026

[5] Koning MV Koning NJ Koning HM van Kleef M. Relationship between sensory stimulation and side effects in percutaneous radiofrequency treatment of the trigeminal ganglion. Pain Pract. (2014) 14:581–7. 10.1111/papr.12124 24152209

[6] Kotecha R Kotecha R Modugula S Murphy ES Jones M Kotecha R. Trigeminal neuralgia treated with stereotactic radiosurgery: the effect of dose escalation on pain control and treatment outcomes. Int J Radiat Oncol Biol Phys. (2016) 96:142–8. 10.1016/j.ijrobp.2016.04.013 27325473

[7] Park SS Lee MK Kim JW Jung JY Kim IS Ghang CG. Percutaneous balloon compression of trigeminal ganglion for the treatment of idiopathic trigeminal neuralgia: experience in 50 patients. J Korean Neurosurg Soc. (2008) 43:186–9. 10.3340/jkns.2008.43.4.186 19096641 PMC2588264

[8] Xu B Jia ZP Ren H Meng L Shen Y Lv R. Preliminarily investigation of the effectiveness of CT-assisted percutaneous microballoon compression treatment for refractory trigeminal neuralgia. Chin J Pain Med. (2019) 25:660–5. 10.3969/j.issn.1006-9852.2019.09.005

[9] Tian DM Zhang JL Duan BB Wang XM Gao XM Ju YX. Analysis and management of abnormal balloon projection shape during percutaneous microballoon compression for the treatment of patients with primary trigeminal neuralgia. Chin J Pain. (2020) 16:51–8. 10.3760/cma.j.issn.2096-8019.2020.01.012

[10] Fang L Tao W Jingjing L Nan J. Comparison of high-voltage- with standard-voltage pulsed radiofrequency of Gasserian ganglion in the treatment of idiopathic trigeminal neuralgia. Pain Pract. (2015) 15:595–603. 10.1111/papr.12227 24954016

[11] Fang L Ying S Tao W Lan M Xiaotong Y Nan J. 3D CT-guided pulsed radiofrequency treatment for trigeminal neuralgia. Pain Pract. (2014) 14:16–21. 10.1111/papr.12041 23433058

[12] Shen Y Meng L Wang T Nan J. The clinical efficacy of high-voltage pulsed radiofrequency on gasserian ganglion for treating idiopathic trigeminal neuralgia. Chin J Pain Med. (2015) 21:38–42. 10.3969/j.issn.1006-9852.2015.01.009

[13] Huo X Sun X Zhang Z Guo W Guan N Luo J. Dyna-CT-assisted percutaneous microballoon compression for trigeminal neuralgia. J Neurointerv Surg. (2014) 6:521–6. 10.1136/neurintsurg-2013-010676 23904451

[14] Xiao X Wei Z Ren H Sun H Luo F. Comparison of Effectiveness and safety between intraoperative 3D-CT-guided and C-arm-guided percutaneous balloon compression for idiopathic trigeminal neuralgia: a multi-center retrospective study. Pain Res Manag. (2021) 2021:9306532. 10.1155/2021/9306532 34194588 PMC8203368

[15] Lin MH Lee MH Wang TC Cheng YK Su CH Chang CM. Foramen ovale cannulation guided by intra-operative computed tomography with integrated neuronavigation for the treatment of trigeminal neuralgia. Acta Neurochir. (2011) 153:1593–9. 10.1007/s00701-011-1009-2 21503836

[16] Aydoseli A Akcakaya MO Aras Y Sabanci PA Unal TC Sencer A. Neuronavigation-assisted percutaneous balloon compression for the treatment of trigeminal neuralgia: the technique and short-term clinical results. Br J Neurosurg. (2015) 29:552–8. 10.3109/02688697.2015.1019418 25807330

[17] Huang B Yao M Wang ZJ Xie KY Shen ZF Fei Y. Clinical and head specimen observation about the position and shape of the balloon in the treatment of patients with trigeminal neuralgia by CT-guided percutaneous microballoon compression under conscious sedation and analgesia. Chin J Pain. (2020) 16:43–50.

[18] Ren YE Han WB Du YM Cong GJ Liu GZ. Efficacy and safety of CT-guided percutaneous microballoon compression in the treatment of patients with primary trigeminal neuralgia under conscious trigeminal ganglion local block. Chin J Pain. (2020) 16:30–5.

[19] Wiggins A Lonie M Pimentil I Newall N Bodkin P Venkatesh A. Electromagnetic neuronavigation for the percutaneous treatment of trigeminal neuralgia with balloon compression: technical note and cadaveric validation study. Acta Neurochir. (2018) 160:1337–41. 10.1007/s00701-018-3548-2 29675717

[20] Montano N Ioannoni E Rapisarda A. The risk of mastication weakness after percutaneous balloon compression for the treatment of trigeminal neuralgia. Clin Neurol Neurosurg. (2020) 195:105880. 10.1016/j.clineuro.2020.105880 32413677

[21] Noorani I Lodge A Vajramani G Sparrow O. The Effectiveness of percutaneous balloon compression, thermocoagulation, and glycerol rhizolysis for trigeminal neuralgia in multiple sclerosis. Neurosurgery. (2019) 85:E684–92. 10.1093/neuros/nyz103 30957177

[22] Zhao ZZ Song MH Jiang F Yu Y Sun MJ Chen FQ. Comparison between radiofrequency thermocoagulation and balloon compression in the treatment of primary trigeminal neuralgia. Chin J Pain. (2021) 17:36–41. 10.3760/cma.j.cn101379-20200114-00007

[23] Ying X Wang H Deng S Chen Y Zhang J Yu W. Long-term outcome of percutaneous balloon compression for trigeminal neuralgia patients elder than 80 years: a STROBE-compliant article. Medicine. (2017) 96:e8199. 10.1097/MD.0000000000008199 28953684 PMC5626327

[24] Wang R Tang YR Chen FQ. Efficacy of short time and repeated percutaneous microballon compression under the guidance of digital subtraction angiography in the treatment of patients with primary trigeminal neuralgia. Chin J Pain. (2020) 16:36–42. 10.3760/cma.j.issn.2096-8019.2020.01.010

[25] Yan XX Zhang SP Quan JJ Zhang X Ren PY Qu JQ. A preliminary study on the intra-balloon pressure monitoring during percutaneous balloon compression in the treatment of trigeminal neuralgia. J Shanxi Med Univer. (2020) 51:588–92. 10.13753/j.issn.1007-6611.2020.06.021

